# Supplementary material for: House Dust Mite Exposure through Human Milk and Dust: What Matters for Child Allergy Risk?
Source: Nutrients. 2022 May 17;14(10):2095. doi: 10.3390/nu14102095 (PMC9143372; doi:10.3390/nu14102095)
Supplement: Supplementary file 1 [file nutrients-14-02095-s001.zip › nutrients-1687214-supplementary.pdf]

Table S1. Associations between having high level of IgE and the presence of Der p 1 in human milk and infant's mattress dust

	Der p 1 < LOD	*Der p* 1 ≥ LOD	Crude		Adjusted	
**Age**	**n/N (%)**	**n/N (%)**	**OR**	**(95% CI)**	**OR**	**(95% CI)**
**Human milk**						
Overall			**1.84**	**(1.06-3.20)**	**1.83**	**(1.05-3.20)**
4	16/83 (19)	11/46 (24)	1.36	(0.62-2.98)	1.28	(0.57-2.86)
8	29/95 (31)	18/50 (36)	1.57	(0.79-3.11)	1.53	(0.76-3.06)
12	22/78 (28)	20/36 (56)	**2.48**	**(1.18-5.19)**	**2.59**	**(1.22-5.47)**
16	8/40 (20)	8/20 (40)	**3.25**	**(1.12-9.41)**	**3.30**	**(1.12-9.73)**
**Infant's mattress dust**						
Overall			1.24	(0.70-2.22)	1.20	(0.65-2.22)
4	16/70 (23)	6/42 (14)	0.77	(0.32-1.85)	0.82	(0.34-2.01)
8	22/78 (28)	22/50 (44)	1.54	(0.76-3.12)	1.55	(0.74-3.27)
12	19/58 (33)	14/39 (36)	1.10	(0.52-2.35)	0.94	(0.43-2.06)
16	5/30 (17)	9/22 (41)	1.88	(0.58-6.15)	1.79	(0.52-6.12)

Prevalence, crude and adjusted ORs for having high levels of serum IgE (IgE > 150 kU/L) in children breastfed by mothers with detectable versus undetectable Der p 1 in human milk and infant's mattress. ORs were adjusted for child's age at the time of asthma reporting, maternal asthma and maternal allergy to house dust mites, sex, and pets in the child's home during the first year of life (same as in Gehring U et al., Allergy. 2012).

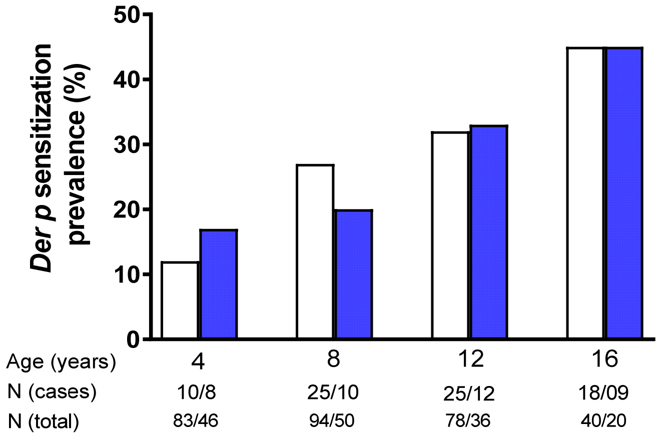

Figure S1: Prevalence of *Der p* sensitization in children according to the presence of *Der p* 1 in human milk. Prevalence of *Der p* sensitization (Der p specific IgE ≥0.35 kU/L) in children from mothers with detectable (blue column) and undetectable (white columns) levels of *Der p* 1 in human milk. N represents the number of children sensitized to *Der p* (cases) and total population (total). Chi-square test was used to compare the prevalence of *Der p* sensitization between groups.
